# Lysosomal Acid Lipase Hydrolyzes Retinyl Ester and Affects Retinoid Turnover*

**DOI:** 10.1074/jbc.M116.724054

**Published:** 2016-06-27

**Authors:** Lukas Grumet, Thomas O. Eichmann, Ulrike Taschler, Kathrin A. Zierler, Christina Leopold, Tarek Moustafa, Branislav Radovic, Matthias Romauch, Cong Yan, Hong Du, Guenter Haemmerle, Rudolf Zechner, Peter Fickert, Dagmar Kratky, Robert Zimmermann, Achim Lass

**Affiliations:** From the ‡ Institute of Molecular Biosciences, University of Graz, 8010 Graz, Austria,; the § Institute of Molecular Biology and Biochemistry and; ¶ Laboratory of Experimental and Molecular Hepatology, Division of Gastroenterology and Hepatology, Department of Internal Medicine, Medical University of Graz, 8010 Graz, Austria,; the ‖ Department of Pathology and Laboratory Medicine and Indiana University Simon Cancer Center, Indiana University School of Medicine, Indianapolis, Indiana 46202, and; ** BioTechMed, Graz 8010, Austria

**Keywords:** hydrolase, lipase, retinoid, retinol, vitamin A, knockout mouse, lysosomal acid lipase, retinyl ester

## Abstract

Lysosomal acid lipase (LAL) is essential for the clearance of endocytosed cholesteryl ester and triglyceride-rich chylomicron remnants. Humans and mice with defective or absent LAL activity accumulate large amounts of cholesteryl esters and triglycerides in multiple tissues. Although chylomicrons also contain retinyl esters (REs), a role of LAL in the clearance of endocytosed REs has not been reported. In this study, we found that murine LAL exhibits RE hydrolase activity. Pharmacological inhibition of LAL in the human hepatocyte cell line HepG2, incubated with chylomicrons, led to increased accumulation of REs in endosomal/lysosomal fractions. Furthermore, pharmacological inhibition or genetic ablation of LAL in murine liver largely reduced *in vitro* acid RE hydrolase activity. Interestingly, LAL-deficient mice exhibited increased RE content in the duodenum and jejunum but decreased RE content in the liver. Furthermore, LAL-deficient mice challenged with RE gavage exhibited largely reduced post-prandial circulating RE content, indicating that LAL is required for efficient nutritional vitamin A availability. In summary, our results indicate that LAL is the major acid RE hydrolase and required for functional retinoid homeostasis.

## Introduction

Lysosomal acid lipase (LAL,^2^ annotated as LIPA) exhibits hydrolytic activity at acidic pH and is known to hydrolyze cholesteryl esters (CEs) and triglycerides (TGs) (1, 2). The essential role of LAL in CE and TG catabolism is evident from the phenotype of LAL-deficient mice and humans (3–5). LAL-deficient mice exhibit a massive accumulation of CEs and TGs in multiple tissues, most prominently in the liver, spleen, and small intestine (4). Moreover, the severity of CE and TG accumulation progresses with age, and mice die at the age of 7–8 months (4). Humans carrying mutations in *LIPA*, which cause partial or complete inactivation of the protein, exhibit phenotypes known as CE storage disease or Wolman disease, respectively (3, 6). These patients also show massive accumulation of CEs and TGs in the liver and small intestine. In contrast to mice, however, complete inactivation of LAL in humans (patients suffering from Wolman disease) leads to an infantile-onset phenotype, and affected individuals usually die within the first month of life. Individuals with residual LAL activity (patients suffering from CE storage disease) show a milder phenotype (similar to that of LAL knockout mice), characterized by CE deposition in visceral tissues, hepatomegaly, and premature death in adulthood.

Despite the severe defect in CE and TG metabolism of LAL-deficient mice and humans, no defect in vitamin A metabolism has been reported. In fact, humans suffering from CE storage disease or Wolman disease show normal plasma retinol (ROH) levels (7). Because intestinal chylomicrons transport TGs, CEs, and also REs, one would expect that a defect in the lysosomal system would not only compromise cholesterol and fatty acid but also vitamin A metabolism.

In this study, we investigated whether REs *per se* are a substrate for LAL. We found that murine LAL (mLAL) hydrolyzes REs. Furthermore, pharmacological inhibition of LAL in the human hepatocyte cell line HepG2 led to an accumulation of REs in endosomal/lysosomal-enriched fractions, suggesting that LAL is limiting for the clearance of endocytosed REs. Unexpectedly, LAL-deficient mice showed decreased RE content in the liver but increased RE accumulation in the duodenum and jejunum. Furthermore, upon RE gavage, LAL-deficient mice exhibited reduced post-prandial RE levels in the circulation, indicating that LAL is required for efficient availability of nutritional vitamin A. Despite compromised vitamin A availability, LAL-deficient mice exhibited elevated circulating ROH levels, arguing against a vitamin A deficiency of these mice.

## Experimental Procedures

### 

#### 

##### Materials

Essentially fatty acid-free BSA was obtained from Sigma-Aldrich (St. Louis, MO). 1,2-diheptadecanoyl-*sn*-glycero-3-phosphatidylcholine, 1,2-dinonadecanoyl-*sn*-glycero-3-phosphatidylcholine, and retinyl acetate were obtained from Larodan Fine Chemicals AB (Stockholm, Sweden) and Sigma-Aldrich and used as internal standards. Retinyl palmitate (RP) and cholesteryl oleate were purchased from Sigma-Aldrich. Lalistat 2 was a kind gift from Dr. Paul Helquist (Department of Chemistry and Biochemistry, University of Notre Dame, Notre Dame, IN).

##### Animals

LAL knockout mice were generated as described previously (4, 5). LAL knockout mice originated from a mixed 129Sv and CF-1 background and were backcrossed onto C57Bl/6 mice for 10 generations. Mice were obtained from a heterozygous breeding stock. Littermates were genotyped by PCR method of the WT and targeted allele as described previously (5). WT and LAL-deficient mice were maintained on a regular light-dark cycle (12 h light, 12 h dark) and kept in a clean environment with *ad libitum* access to food (Altromin 1324, 4% fat and 19% protein, 15,000 IU vitamin A/kg (as retinyl acetate), Altromin Spezialfutter GmbH & Co. KG, Lage, Germany) and water. Age-matched, 3-, 6.5-, and 8-month-old male and female mice were used for all experiments. Mice were sacrificed by cervical dislocation. All experiments were approved by the Federal Ministry of Science, Research, and Economy and local ethic committees and were in accordance with the Council of Europe Conventions.

##### cDNA Cloning of Recombinant His-tagged Proteins

Poly(A)+RNA was isolated from murine liver using the Oligotex mRNA mini kit from Qiagen GmbH (Hilden, Germany). Liver mRNA was transcribed into first-strand cDNA using the SuperScript® reverse transcriptase protocol (Invitrogen). Second-strand cDNA was obtained by addition of *Escherichia coli* DNA ligase buffer, *E. coli* DNA polymerase, *E. coli* DNA ligase (all chemicals from New England Biolabs, Inc., Beverly, MA), and deoxyribonucleotide triphosphates (Carl Roth GmbH and Co. KG, Karlsruhe, Germany) to the mixture and subsequent incubation at 16 °C for 3 h. Thereafter, T4 DNA polymerase (New England Biolabs, Frankfurt am Main, Germany) was added and further incubated for 20 min to give blunt-end cDNA. The coding sequences were amplified by PCR from liver cDNA using Phusion® DNA polymerase (New England Biolabs). Cloning of mHSL into the mammalian expression vector pcDNA4/HisMax C (Invitrogen) has been described previously (8). The mLAL coding sequence was subcloned into the pcDNA4/HisMax expression vector by overlap extension PCR to obtain a C-terminal His-tagged fusion protein instead of the N-terminal His tag of the vector. In brief, the upstream part of the vector backbone without the His tag and the mLAL coding sequence were PCR-amplified from the pcDNA4/HisMax C plasmid containing the mLAL coding sequence. Overlapping regions and the 3′ end-located His tag were introduced by primers. The newly synthesized PCR fragment and the expression plasmid pcDNA4/HisMax C were digested with SacI/XhoI and ligated. The following primers were used: SP163fw (SacI), 5′ GCC GAG CTC TAA TAC GAC TCA CTA TAG GGA GAC 3′; SP163/LALrv, 5′ GAA CAC CAG GCC CTG GAG TTG CAT GGT TTC GGA GGC CGT CCG G 3′; SP163/LALfw, 5′ C CGG ACG GCC TCC GAA ACC ATG CAA CTC CAG GGC CTG GTG TTC 3′; and LALrv (XhoI), 5′ CTC GAG TCA ATG ATG ATG ATG ATG ATG CTG GTA TTT CTT CAT TAG ACT GAT 3′. A control pcDNA4/HisMax vector encoding β-galactosidase (LacZ) was provided by the manufacturer (Invitrogen).

##### Expression of Recombinant Proteins and Preparation of Cell Lysates

Monkey embryonic kidney cells (COS-7, ATCC, CRL-1651^TM^) were cultivated in DMEM (Gibco, Invitrogen) containing 10% fetal calf serum (Sigma-Aldrich) and antibiotics at 37 °C in humidified air (89–91% saturation) and 5% CO_2_. Cells were seeded in 10-cm dishes at a density of 900,000 cells/dish. The following day, cells were transfected with plasmid DNA encoding the respective His-tagged recombinant proteins or LacZ as a control using Metafectene® (Biontex, Munich, Germany) as a transfection reagent. Cells were harvested 2 days after transfection using a cell scraper. Cells were lysed on ice in solution A (0.25 m sucrose, 1 mm EDTA, 1 mm DTT, 20 μg/ml leupeptine, 2 μg/ml antipain, and 1 μg/ml pepstatin (pH 7.0)) by sonication (Virsonic 475, Virtis, Gardiner, NJ). Nuclei and unbroken cells were removed by centrifugation at 1000 × *g* and 4 °C for 5 min, and lysates were stored at −20 °C until further use. Expression of His-tagged proteins was assessed by Western blotting.

##### Measurement of in Vitro RE Hydrolase Activity

For assessment of RE hydrolase activity of recombinant proteins, 100 μl of COS-7 cell lysates (100 μg of cell protein) containing overexpressed recombinant proteins and 100 μl of substrate were incubated at 37 °C for 60 min. The reaction was terminated by addition of 100 μl of methanol containing 1.5 μm retinyl acetate as internal standard and 1 ml of hexane. Then the samples were vigorously vortexed and centrifuged at 5000 × *g* for 5 min at 4 °C. The upper hexane phase (800 μl) was collected and dried under nitrogen. Samples were dissolved in 200 μl of chloroform/methanol (2/1, v/v) and analyzed by HPLC fluorescence detection (FD) (see “Preparation of Tissue Homogenates and Quantification of Neutral Lipids by HPLC FD”). Blank incubation was performed with 100 μl of solution A under identical conditions and used for background correction. The RE substrate contained 600 μm retinyl palmitate and was emulsified by sonication (Virsonic 475, Virtis) with 300 μm phosphatidylcholine/phosphatidylinositol (PC/PI, 3/1, M/M) in 100 mm potassium phosphate buffer (pH 7.4) or 100 mm sodium acetate buffer (pH 4.5), respectively. In some cases, substrates also contained increasing concentrations of BMP (0–500 μm).

For the measurement of liver acid RE hydrolase activity, perfused liver sections of *ad libitum*-fed mice, 3 month of age, were homogenized in solution A using an UltraTurrax (IKA, Janke & Kunkel, Germany) and adjusted to a protein concentration of 2 mg/ml. Then 75 μl of the prepared lysates (150 μg of protein) was delipidated, and proteins were precipitated by addition of 600 μl of ice-cold acetone. Thereafter, precipitated proteins were pelleted by centrifugation at 20,000 × *g* at 4 °C for 30 min. Pelleted proteins were dried under a stream of nitrogen gas and dissolved in 100 μl of solution A. For quantification of RE hydrolase activity, 100 μl of substrate was added and incubated for 60 min at 37 °C. Blank incubations were performed with 100 μl of solution A under identical conditions and 100 μl of delipidated liver proteins with sodium acetate buffer (pH 4.5) without substrate to correct for residual endogenous ROH release. The substrate contained 600 μm retinyl palmitate and was emulsified (as above) in 100 mm sodium acetate buffer (pH 4.5) containing 300 μm PC/PI (3/1, M/M) and 300 μm BMP.

##### Measurement of in Vitro CE and TG Hydrolase Activity

The TG hydrolase activity assay was performed as described previously (8) with some modifications. In brief, 100 μl of COS-7 cell lysates (30 μg of cell protein) was incubated with 100 μl of substrate for 60 min at 37 °C. Blank incubation was performed with 100 μl of solution A under identical conditions and used for background correction. Reactions were terminated by addition of 3.25 ml of methanol/chloroform/*n*-heptane (10/9/7, v/v/v) and 1 ml 0.1 m potassium carbonate (pH 10.5). After vigorous mixing, phase separation was achieved by centrifugation at 1000 × *g* for 10 min. The radioactivity in 200 μl of the upper aqueous phase was determined by liquid scintillation counting. The TG substrate contained 1.67 mm triolein and 1.25 μCi [^3^H]triolein (per reaction) and was emulsified with 300 μm PC/PI (3/1, M/M) and 300 μm BMP in 100 mm potassium phosphate buffer (pH 7.4) or 100 mm sodium acetate buffer (pH 4.5), respectively.

The CE hydrolase activity assay was performed as described above for the TG hydrolase activity assay. The CE substrate contained 1 mm cholesteryl oleate and 1.25 μCi [^3^H]cholesteryl oleate (per reaction) and was emulsified with 90 μm PC/PI (3/1, M/M) and 90 μm BMP in 100 mm potassium phosphate buffer (pH 7.4) or 100 mm sodium acetate buffer (pH 4.5), respectively.

##### Preparation of RE-enriched Lipoproteins

Blood (200 ml) was collected from a 29-year-old healthy male volunteer, and EDTA (5 mm) plasma was prepared. Isolation of TG-rich lipoproteins was performed according to a standard protocol. In brief, plasma was adjusted to a density of *d* = 1.21 using potassium bromide and overlaid with a discontinuous salt gradient (sodium chloride) with densities of *d* = 1.063, *d* = 1.019, and *d* = 1.006. Samples were centrifuged for 24 h at 40,000 rpm and 4 °C using a Beckman Optima L-90K centrifuge and a SW41 rotor (Beckman Instruments Inc., Palo Alto, CA). After centrifugation, TG-rich lipoproteins mostly consisting of chylomicrons and chylomicron remnants were collected from the top of the tube and stored at −20 °C for further use. For RE enrichment, 300 μl of isolated lipoproteins was mixed with 40 μmol of retinyl palmitate (Sigma-Aldrich) and brought to a total volume of 2 ml using 10 mm phosphate-buffered saline. Then the mixture was sonicated three times for 30 s with an amplitude of 1 (Virsonic 475, Virtis).

##### Cultivation of HepG2 Cells and Loading with Human TG-rich Lipoproteins

HepG2 cells (ATCC, HB-8065^TM^) were seeded in Greiner CELLSTAR® 6-well cell culture plates (Sigma-Aldrich) and incubated with DMEM (Gibco, Invitrogen), supplemented with 10% fetal calf serum and antibiotics, under standard cell culture conditions. To inhibit LAL activity, cells were incubated with 10 μm LAL inhibitor Lalistat 2 for 72 h as described previously (11). As a control, cells were incubated with DMSO. Thereafter, RE-enriched lipoproteins were added to the incubation medium to give a final concentration of 10 μm RE, and cells were further incubated for 6 h. Cells were washed twice with Dulbecco's phosphate-buffered saline and harvested using a cell scraper, and intracellular retinoid content was analyzed by HPLC FD as described below.

##### Isolation of LAMP1/RAB7-rich Fractions

HepG2 cells were harvested using a cell scraper and disrupted in buffer A containing 30% iodixanol (OptiPrep^TM^, Axis-Shield PoC AS, Kjelsåsveien, Norway) using a standard Dounce homogenizer. Disrupted cells were transferred to a centrifugation tube and overlaid with a discontinuous iodixanol gradient (prepared in solution A) with densities of 20%, 15%, 10%, and 0% iodixanol. Samples were centrifuged for 2 h at 40,000 rpm and 4 °C using a Beckman Optima L-90K centrifuge and an SW41 rotor (Beckman Instruments Inc.). Bands were collected from the top of each density interphase (F1, 0–10%; F2, 10–15%; F3, 15–20%) and analyzed by Western blotting analysis using ras-associated protein 7 (Rab7)/lysosomal-associated membrane protein 1 (LAMP1) and protein disulfide isomerase as marker proteins for endosomes/lysosomes and the endoplasmic reticulum, respectively.

##### Preparation of Tissue Homogenates and Quantification of Neutral Lipids by HPLC FD

Various tissues (100–300 μg of protein) of *ad libitum*-fed LAL knockout mice and WT littermates were homogenized in solution A using an UltraTurrax (IKA, Janke & Kunkel). Lipids were Folch-extracted. Briefly, lipids were extracted twice with chloroform/methanol (2/1, v/v) containing 1% acetic acid and 500 nm butylated hydroxyl toluene. Lipid extracts were dried under a stream of nitrogen and stored at −20 °C. Prior to analysis, dried lipids were dissolved in chloroform/methanol (2/1, v/v). TG, PC, free cholesterol, and CE were separated on a Betasil® diol column (100 × 4.6 mm, 5 μm, Thermo Fisher Scientific, Waltham, MA) using a ternary gradient solvent system and detected by an HPLC evaporative light scattering detector (16). The HPLC evaporative light scattering detector consisted of a precooled sample manager (at 4 °C), pump, injector, and column oven (at 40 °C), all of the 1100 series (Agilent, Santa Clara, CA), and were coupled to a Sedex 85 evaporative light scattering detector (Sedere, Alfortville, France). Data were analyzed using Chemstation software (B 04.01, Agilent). Neutral lipid standards were prepared as 1 mg/ml stock solutions in chloroform/methanol (2/1, v/v). Calibration curves were measured from 2.7–350 μg/ml.

For retinoid analyses, tissue homogenates were mixed with an equal amount of methanol containing 1.5 μm all-trans retinyl acetate (Sigma-Aldrich) as internal standard. Retinoids were extracted twice using hexane, as described previously (16). Organic phases were combined and dried under a stream of nitrogen and dissolved in chloroform/methanol (2/1, v/v). Retinoids were separated on a YMC-Pro C18 (150 × 4.6 mm, S-5 μm, 12 nm, YMC Europe GmbH, Dinslaken, Germany) using a gradient solvent system (flow, 1 ml/min; gradient, 1–5 min 100% methanol, 5–14 min 60%/40% methanol/toluene, and 14–18 min 100% methanol) and detected at excitation 325 nm/emission 450 nm. The HPLC consisted of a Waters e2695 separation module, including a column oven (at 25 °C) and a Waters 2475 fluorescence detector (Waters Corp., Milford, MA). Data were analyzed using Empower 3 chromatography data software (Waters Corp.).

##### Measurement of Plasma ROH and Retinol Binding Protein 4 (RBP4) levels

Blood of *ad libitum*-fed 8-month-old female mice was collected, and EDTA plasma was prepared. For determination of ROH levels, 40 μl of plasma was mixed with 100 μl of methanol containing retinyl acetate (1.5 μm) as internal standard, extracted into hexane, and analyzed by HPLC FD (as above). Plasma RBP4 levels were assessed by Western blotting.

##### Administration of Oral RE Gavage and Measurements of Post-prandial Plasma Retinoid Levels

Female mice, 8 months of age, were weighed and received gavage of 300 IU of vitamin A equivalents (retinyl palmitate in olive oil) per gram of bodyweight using a standard gavage needle. Before and 2 h after gavage, blood samples were collected from the vena facialis, and EDTA plasma was prepared. For retinoid measurements, 40 μl of plasma was extracted into hexane and analyzed using HPLC FD (as above).

##### Analysis of mRNA Expression by Quantitative Real-time PCR

Total RNA of livers was extracted and reverse-transcribed into cDNA. Quantitative real-time PCR for lecithin:retinol acyltransferase (LRAT) was performed as described previously (8). Target gene expression was calculated by the ΔΔCt method using the ribosomal gene 36B4 as a housekeeping gene. The following primer pairs for detection of LRAT and 36B4 mRNA were used, respectively: LRAT-fw, 5′ CCG TCC CTA TGA AAT CAG CTC 3′; LRAT-rev, 5′ ATG GGC GAC ACG GTT TTC C 3′; 36B4-fw, 5′ GCT TCA TTG TGG GAG CAG ACA 3′; and 36B4-rev, 5′ CAT GGT GTT CTT GCC CAT CAG 3′.

##### Western Blotting Analysis

Proteins of cell extracts, tissue homogenates, and murine plasma (1/40 dilution) were separated by 10% SDS-PAGE and blotted onto polyvinylidene fluoride membranes (Carl Roth GmbH, Karlsruhe, Germany). The following primary antibodies were used: N-terminal His_6_ tag (27-4710-01, Amersham Biosciences, Buckinghamshire, UK), C-terminal His_6_ tag (102-PA80, Reliatech, Wolfenbüttel, Germany), and RBP4 (sc-25850, Santa Cruz Biotechnology Inc., Dallas, TX) as well as protein disulfide isomerase, LAMP1, and RAB7 from Cell Signaling Technology (35019, C54H11, and 2094S, respectively, Cambridge, UK). After binding of the secondary anti-mouse or anti-rabbit horseradish peroxidase-labeled antibody, signals were visualized by ECL detection (ECL Plus/Prime, GE Healthcare). For densitometric analysis, intensities of RBP4 bands were normalized to the corresponding intensities of the protein stain on the membrane using ImageJ software (freely available at http://imagej.nih.gov/ij/).

##### Statistical Analysis

Data are mean + S.D. Statistically significant differences were determined by Student's unpaired *t* test (two-tailed). Group differences were considered statistically significant for *p* < 0.05 (*), *p* < 0.01 (**), and *p* < 0.001 (***).

## Results

### 

#### 

##### Lysosomal Acid Lipase Hydrolyzes REs

**FIGURE 1.**
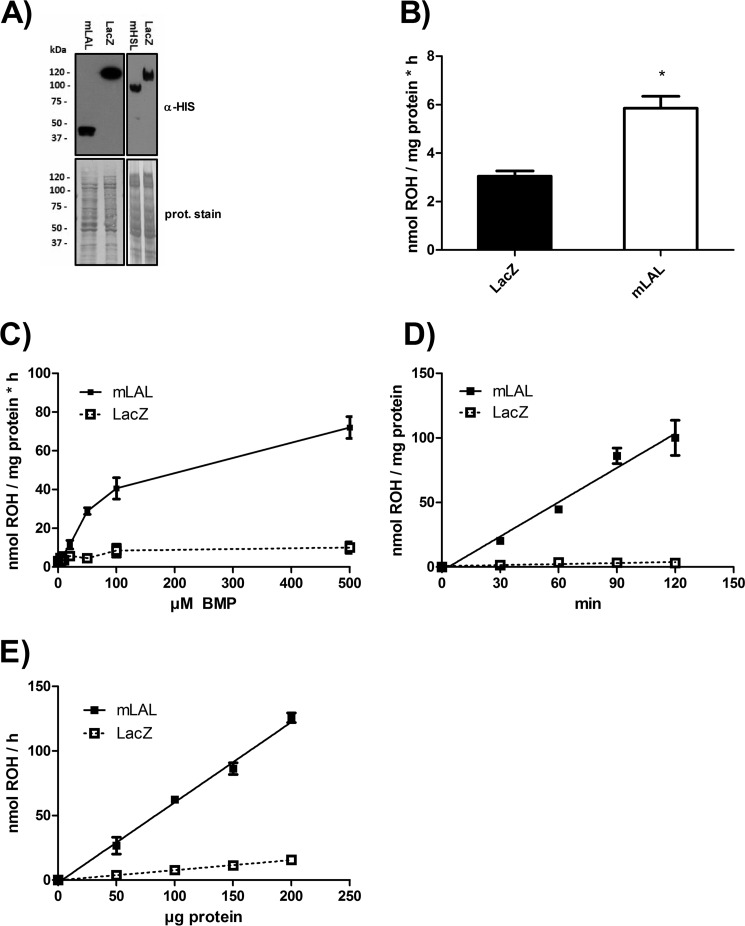
**mLAL exhibits RE hydrolase activity, which is stimulated by BMP.** COS-7 cells were transfected with pcDNA4/HisMax plasmids encoding mLAL and β-galactosidase (LacZ) as a control. After 48 h, cells were collected, and lysates (1000 × *g* supernatant) were prepared. *prot*, protein. *A*, expression of His-tagged proteins was verified by Western blotting using α-His antibody. *B*, lysates were incubated with RP emulsified with PC/PI in sodium acetate (pH 4.5). Lipids were extracted into hexane, and ROH content was quantified by HPLC. *C*, as in *B*, but increasing concentrations of BMP were added to the substrate. *D* and *E*, lysates were incubated for various times as indicated (*D*), or various amounts of lysates were incubated for 1 h with substrates as in *A* but containing 300 μm BMP (*E*). Then the ROH content was determined by HPLC FD, and the rates of hydrolysis were calculated. Data are mean ± S.D. of triplicate determination and are representative of three independent determinations. Statistically significant differences were determined in comparison with control LacZ by Student's unpaired *t* test (two-tailed). *, *p* < 0.05.

To determine whether LAL exhibits hydrolytic activity against REs as substrate, we expressed recombinant mLAL or LacZ (control) in COS-7 cells. Fig. 1*A* depicts Western blotting analyses of recombinant proteins. Incubation of mLAL containing lysates with RP as substrate at (pH 4.5) led to 1.9-fold increased hydrolytic activity compared with control lysates (expressing LacZ) (Fig. 1*B*). Because LAL is known to be activated by anionic phospholipids such as BMP (9), we added increasing concentrations of BMP to the substrate. Addition of BMP led to a dose-dependent increase of RE hydrolase activity of mLAL-containing lysates, resulting in ∼7-fold higher activity at 500 μm BMP compared with control lysates (Fig. 1*C*). To validate the RE hydrolase activity of mLAL, we performed activity assays with increasing incubation times and amounts of lysates. As expected, we observed that, under our assay conditions, mLAL hydrolyzed RP (in the presence of 300 μm BMP) in a time- (Fig. 1*D*) and dose-dependent (Fig. 1*E*) manner.

**FIGURE 2.**
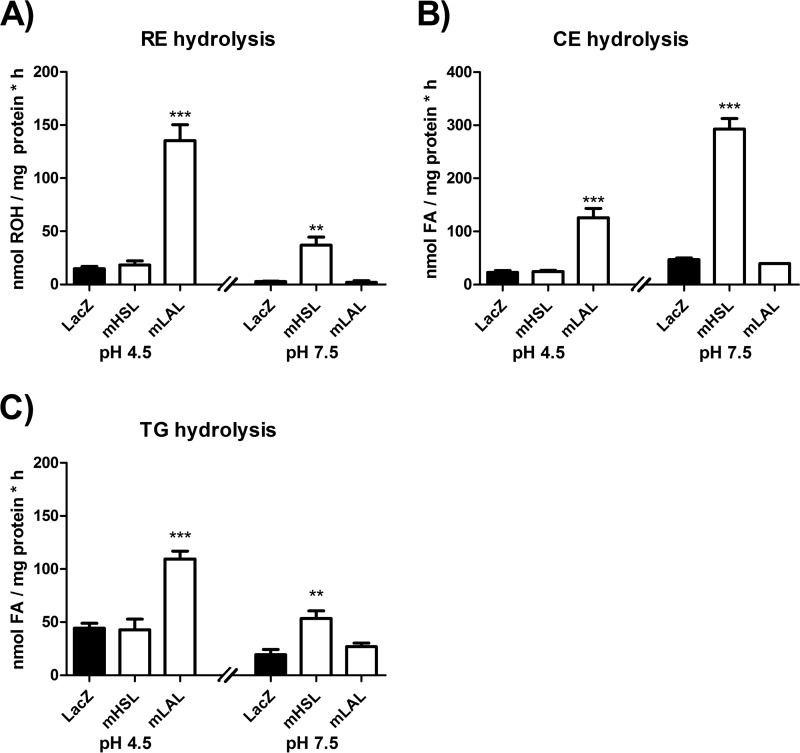
**mLAL hydrolyzes RE, CE, and TG substrates at comparable rates.** *A–C*, lysates were incubated with either retinyl palmitate (*A*), [^3^H]cholesteryl oleate (*B*), or [^3^H]triolein (*C*) as substrates in sodium acetate buffer (pH 4.5) or potassium phosphate buffer (pH 7.5) containing 300 μm BMP. Then the release of ROH (*A*) or ^3^H fatty acids (*FA*, *B* and *C*) was determined by HPLC FD or scintillation counting, respectively. Rates were normalized to milligram of protein. Data are mean ± S.D. of triplicate determination and are representative of three independent determinations. Statistically significant differences were determined in comparison with control LacZ by Student's unpaired *t* test (two-tailed). **, *p* < 0.01; ***, *p* < 0.001.

Next we compared the RE hydrolase activity of lysates containing mLAL with lysates containing murine hormone-sensitive lipase (mHSL), a known RE hydrolase (10). Because the enzymes are active at different pH values, we performed activity assays at acidic and neutral pH. We found that lysates containing mLAL or mHSL showed ∼9- and 13-fold increased hydrolytic activities against RP as substrate at pH 4.5 or 7.5, respectively (Fig. 2*A*). As expected, no activity was observed for mLAL at pH 7.5 and mHSL at pH 4.5. Furthermore, mLAL and mHSL exhibited hydrolytic activities against cholesteryl oleate and triolein as substrates. (Fig. 2, *B* and *C*). Together, the results of the *in vitro* activity assays demonstrate that mLAL hydrolyzes REs and that this activity is strongly stimulated in the presence of BMP.

##### LAL Is Required for Lysosomal RE Clearance

**FIGURE 3.**
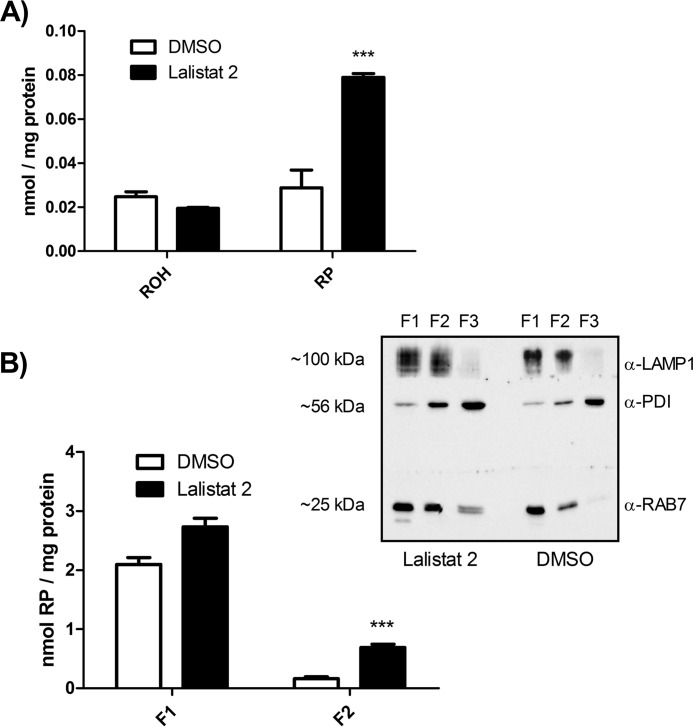
**Inhibition of LAL leads to accumulation of REs in LAMP1/RAB7-enriched fractions of HepG2 cells.** HepG2 cells were incubated with DMEM containing 10 μm Lalistat 2 or a solvent control (DMSO) for 72 h. Then the media were replaced with media containing artificially RP-enriched human TG-rich lipoproteins (final concentration, 10 μm RP) and 10 μm Lalistat 2 or DMSO. After 6 h of incubation, cell lysates were prepared, and endosomal/lysosomal fractions were isolated by density ultracentrifugation. Lipids of cell lysates (*A*) and endosomal/lysosomal fractions (*B*, *F1* and *F2*) were extracted into hexane, and retinoids (ROH and RP) were analyzed by HPLC FD. Retinoid levels were normalized to milligram of protein. Data are mean ± S.D. of triplicate determinations and representative of three independent experiments. Statistically significant differences were determined between treatment groups by Student's unpaired *t* test (two-tailed). ***, *p* < 0.001. *B*, *inset*, enriched fractions (*F1–F3*) were analyzed by Western blotting using LAMP1/RAB7 and protein disulfide isomerase (*PDI*) as endosomal/lysosomal and endoplasmic reticulum marker proteins, respectively.

To investigate whether LAL plays a role in intracellular RE metabolism, we used the human hepatocyte cell line HepG2 and the LAL-specific inhibitor Lalistat 2 (11). We reasoned that inhibition of LAL may impair the lysosomal clearance of REs and, thus, that cells may show increased RE accumulation. We incubated cells with human TG-rich lipoproteins (artificially enriched with RP) in the absence (DMSO control) or presence of Lalistat 2. Analysis of cellular retinoid content revealed that cells incubated with Lalistat 2 exhibited increased RP content (2.5-fold), whereas ROH levels were similar to that of control cells (Fig. 3*A*). To determine whether RE accumulated in the endosomes/lysosomes, we enriched these fractions from cells incubated with Lalistat 2 or DMSO (control) by density gradient centrifugation. Endosomal/lysosomal fractions were identified as LAMP1/RAB7-positive fractions by Western blotting (Fig. 3*B*, *inset*). Retinoid analyses revealed that the LAMP1/RAB7-enriched fraction F1 showed a trend toward increased Res, whereas fraction F2 showed an ∼3-fold increased RE content in cells treated with Lalistat 2 compared with control cells (DMSO) (Fig. 3*B*). In summary, these results suggest that LAL is required for efficient lysosomal clearance of REs.

##### LAL Is the Major Acid RE Hydrolase in Murine Liver

**FIGURE 4.**
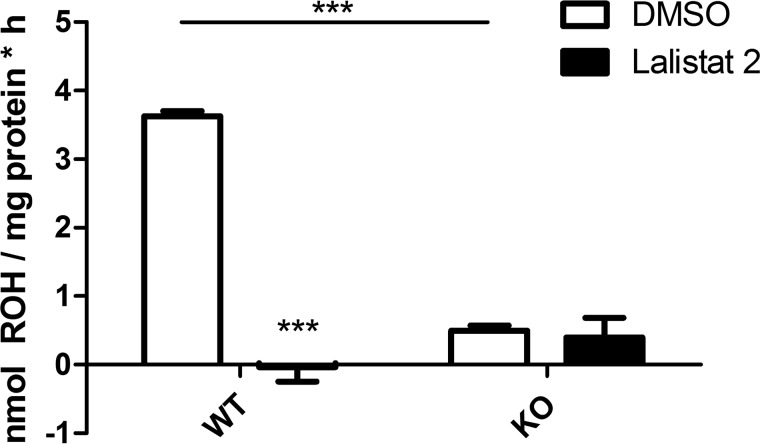
**LAL is responsible for acid RE hydrolase activity in the liver.** Livers of 6.5-month-old, *ad libitum*-fed LAL KO mice and WT littermates were excised, and tissue homogenates were prepared. Proteins of liver homogenates were precipitated and delipidated with acetone. Then proteins were dissolved in solution A, incubated with RP, and emulsified with PC/PI as substrate in sodium acetate buffer (pH 4.5) containing 300 μm BMP and in the absence (DMSO) or presence of 10 μm Lalistat 2 for 60 min. Then lipids of reaction mixtures were extracted, and the ROH content was determined by HPLC FD. Rates of ROH release were normalized to milligram of protein. Data (*n* = 3) are mean ± S.D. of triplicate determinations and are representative of two independent experiments. Statistically significant differences were determined between groups (DMSO and Lalistat 2 and as indicated by the *line*) by Student's unpaired *t* test (two-tailed). ***, *p* < 0.001.

To assess to which extent LAL contributes to acid RE hydrolase activity in tissues, we precipitated and delipidated proteins of liver homogenates from WT and LAL-deficient mice using acetone. Then proteins were reconstituted in aqueous solution and incubated with RP as substrate. To assess LAL-dependent activity, we also added Lalistat 2 to the incubation mixture. We found that liver protein preparations of LAL-deficient mice exhibited 85% reduced acid RE hydrolase activity compared with liver preparations of WT mice (Fig. 4). Addition of Lalistat 2 to the assay mixtures completely inhibited acid RE hydrolase activity in liver preparations of WT mice, whereas no effect was observable in liver preparations of LAL-deficient mice (Fig. 4). These results clearly indicate that LAL is responsible for the majority of acid RE hydrolase activity in the liver.

##### LAL-deficient Mice Exhibit Decreased RE Content in the Liver

**FIGURE 5.**
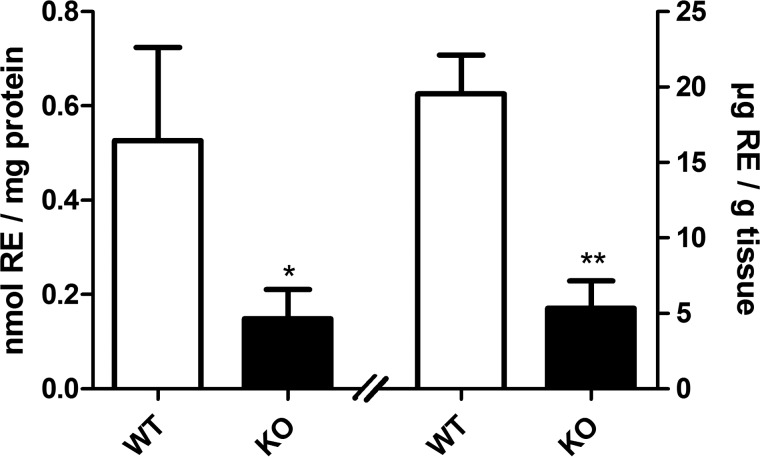
**LAL-deficient mice exhibit reduced RE levels in the liver.** Livers of 3-month-old, *ad libitum*-fed LAL KO mice and WT littermates were excised, and tissue homogenates were prepared. Lipids of homogenates were extracted into hexane, and RP content was quantified by HPLC FD. RP contents were normalized to milligram of protein of homogenates and to gram of wet tissue weight and expressed as nanomoles of RE per milligram of protein (*left graph*) or micrograms of RE per gram of tissue (*right graph*), respectively. Data are presented as mean ± S.D. (*n* = 4 for each genotype). Statistically significant differences were determined between genotypes by Student's unpaired *t* test (two-tailed). *, *p* < 0.05; **, *p* < 0.01.

To get first insights into the physiological role of LAL in RE turnover, we determined ROH and RE content in livers of 3-month-old WT and LAL-deficient mice. Against expectations, we found that livers of LAL-deficient mice exhibit ∼70% decreased RE content compared with that of WT littermates, irrespective of being normalized to milligram of protein of tissue homogenate (Fig. 5, *left half*) or to gram of tissue weight (Fig. 5, *right half*). Decreased retinoid content in livers of LAL-deficient mice may be caused by decreased deposition of REs in the liver because of impaired vitamin A supply.

##### LAL-deficient Mice Accumulate REs in the Duodenum and Jejunum

**FIGURE 6.**
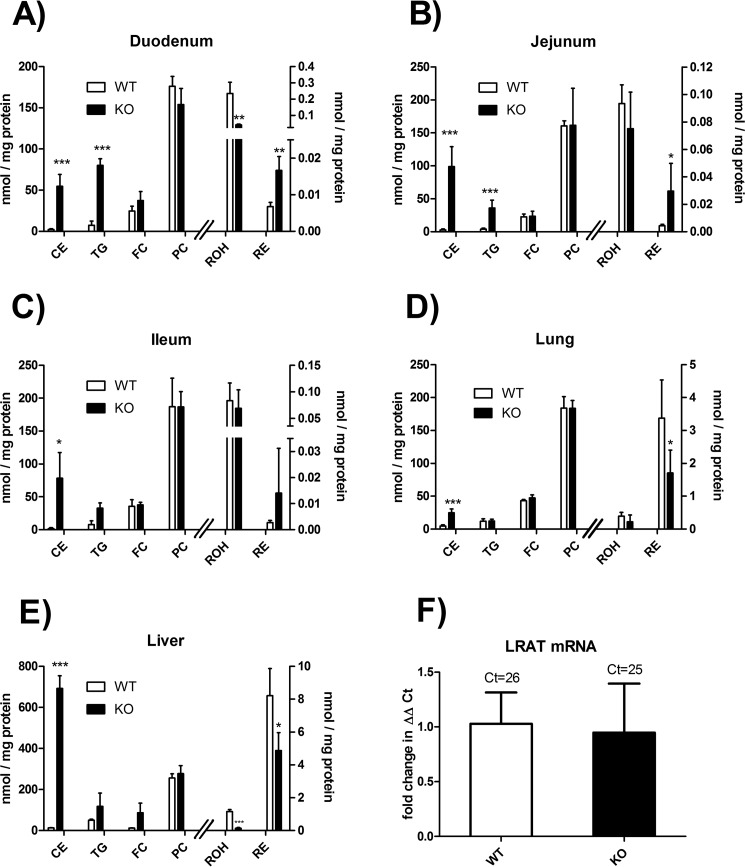
**LAL-deficient mice accumulate RE in the intestine.** Small intestine (*A*, duodenum; *B*, jejunum; *C*, ileum), lung (*D*), and liver (*E* and *F*) of 6.5-month-old, *ad libitum*-fed LAL KO mice and WT littermates were excised, tissue homogenates were prepared (*A–E*), and liver mRNA was isolated (*F*). Lipids of homogenates were extracted. CEs, TGs, free cholesterol (*FC*), and PCs were quantified by HPLC evaporative light scattering detector. For analysis of retinoid content, lipids of homogenates were extracted into hexane, and ROH and RE (RP + RS) were quantified by HPLC FD. Lipid contents were normalized to milligram of protein of homogenates. *F*, LRAT mRNA expression in livers was determined by quantitative real-time PCR and expressed as ΔΔCt using the ribosomal gene 36B4 as a housekeeping gene. Data are presented as mean ± S.D. (*n* = 4–6 for each genotype). Statistically significant differences were determined between genotypes by Student's unpaired *t* test (two-tailed). *, *p* < 0.05; **, *p* < 0.01; ***, *p* < 0.001.

The lipid phenotype of LAL-deficient mice is known to progress with age (4, 12). Because we speculated that this may also be the case for disturbances in vitamin A homeostasis, we measured neutral lipid (including RE) and phospholipid contents in various tissues of 6.5-month-old LAL-deficient mice and WT littermates. The contents of the main phospholipid class, PC, were comparable between WT and LAL-deficient mice in all investigated tissues (Fig. 6). More importantly, and in accordance with previous reports (4, 5, 12), CE content was increased manyfold (up to 50-fold) in all investigated tissues of LAL-deficient mice (Fig. 6, *A–E*). In contrast, free cholesterol concentration was virtually unchanged (Fig. 6, *A–E*). TG and RE contents were significantly increased in the duodenum and jejunum, whereas a trend toward increased TG and RE content was observed in the ileum (Fig. 6, *A–C*). Increased RE content in the duodenum of LAL-deficient mice was accompanied by decreased ROH levels (Fig. 6*A*). In the lung of LAL-deficient mice, CE content was increased whereas that of REs was decreased. All other lipid classes in the lung were comparable between both genotypes (Fig. 6*D*). In accordance with a previous report (4), we observed an enlarged mesenteric lymph node in LAL-deficient mice that contained ∼0.05 and ∼0.6 nmol/mg protein ROH and RP, respectively. Notably, the spleen of LAL-deficient mice contained largely increased RE content (93 ± 36 pmol/mg of protein *versus* 0.17 ± 0.15 pmol/mg of protein in LAL-deficient *versus* WT mice, respectively). Interestingly, and similar to what was already observed in 3-month-old LAL-deficient mice, 6.5-month-old LAL-deficient mice also exhibited reduced ROH and RE content in the liver (Fig. 6*E*). Because livers of LAL-deficient mice exhibited unchanged LRAT mRNA expression (Fig. 6*F*), decreased hepatic RE content was apparently not a consequence of a down-regulation of LRAT expression. Thus, decreased RE content in the liver and lung of LAL-deficient mice, which was accompanied by an accumulation of REs in the duodenum and jejunum, rather suggests that the nutritional supply with vitamin A in these mice might be compromised.

##### LAL-deficient Mice Exhibit Elevated Circulating ROH:RBP4 but Decreased Post-prandial RE Levels

**FIGURE 7.**
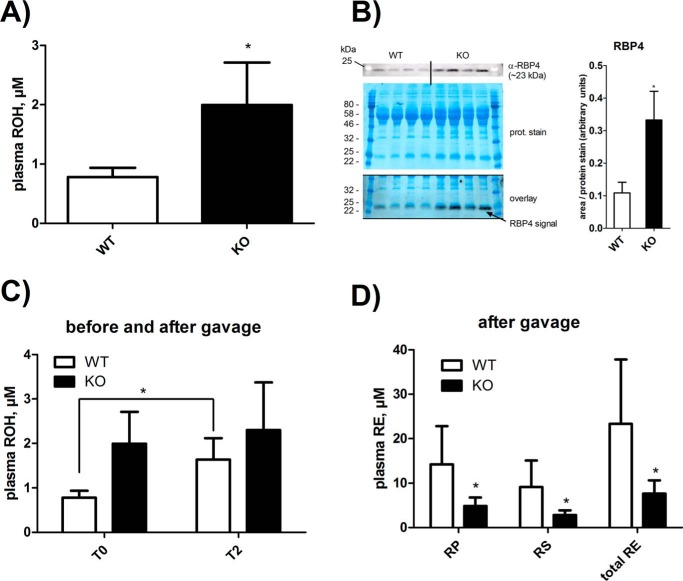
**LAL-deficient mice exhibit increased circulating ROH but decreased post-prandial RE contents.** *A*, blood of 8-month-old, *ad libitum*-fed, age- and gender-matched LAL-deficient mice (*KO*) and WT littermates was collected, and EDTA plasma was prepared. Lipids were extracted into hexane, and ROH content was quantified by HPLC FD. *B*, plasma proteins were separated by SDS-PAGE and immunoblotted using anti-RBP4 antibody (α*-RBP4*). Proteins on the membrane were stained with Coomassie Blue and overlaid with the Western blotting signal. For densitometric analyses, intensities of α-RBP4 bands were normalized to the intensities of the corresponding Coomassie protein stains of the membrane using ImageJ software. *C* and *D*, mice as in *A* received a gavage of 300 IU of vitamin A equivalents RP in olive oil/gram body weight. Before (*T0*) and 2 h after (*T2*) vitamin A gavage, blood was collected, and EDTA plasma was prepared. Lipids of plasma were extracted into hexane. ROH (*C*, for time T0 and T2) and RE (*D*, RP + RS, for time T2) were determined by HPLC FD. Data are expressed as mean ± S.D. (*n* = 4–6 for each genotype) and are representative of two independent experiments. Statistically significant differences were determined between genotypes by Student's unpaired *t* test (two-tailed). *, *p* < 0.05.

To investigate whether accumulation of RE in the duodenum and jejunum affects circulating vitamin A availability, we measured ROH concentrations in the plasma of *ad libitum*-fed mice. Interestingly, we found that circulating ROH levels of LAL-deficient mice were 2.5-fold higher compared with those of WT mice (Fig. 7*A*). To examine whether increased plasma ROH levels were accompanied by increased levels of its specific transport protein, RBP4, we performed Western blotting analyses using an antibody specific against RBP4 (Fig. 7*B*). Loading equal amounts of protein, we found more intense bands in plasma samples of LAL-deficient mice at a molecular weight close to 25 kDa compared with that of control mice. An overlay of the Western blotting signal with the protein stain of the membrane showed that the α-RBP4 band located right below the intense protein band at 25 kDa, suggesting that the α-RBP4 signal was apparently due to a specific interaction with RBP4 protein (∼23 kDa). Densitometric quantitation revealed a 3-fold increase in the RBP4 signal of the Western blot, which is in line with increased ROH levels in the plasma of LAL-deficient mice.

To investigate whether LAL deficiency affects the nutritional availability of vitamin A, we performed gavage with an RP:olive oil bolus and measured post-prandial circulating retinoid levels. Interestingly, after 2 h of RP gavage (T2), LAL-deficient mice exhibited virtually unchanged plasma ROH levels, whereas those of WT mice increased by 2.1-fold (Fig. 7*C*). Before RP gavage, plasma RP was not detectable in both genotypes. 2 h after RP gavage, LAL-deficient mice exhibited lower plasma RP and retinyl stearate (RS) concentration, which in total corresponded to 70% lower plasma RE content compared with that of WT mice (Fig. 7*D*). Together, the observations that, upon RP gavage, the plasma ROH content of LAL-deficient mice did not increase and that their plasma RE content was markedly reduced demonstrate that LAL is required for efficient nutritional availability of vitamin A. However, LAL is not limiting for circulating ROH:RBP4 levels, and, if absent, it does not provoke vitamin A deficiency.

## Discussion

This study was prompted by the following observations: endocytosed chylomicron REs are predominantly cleared in the early/late endosome and not in lysosomes (13, 14); two different enzymes are thought to be responsible for acid RE and CE hydrolase activity (15); and LAL-deficient mice accumulate TGs and CEs in various tissues, but RE levels have not been reported. Because it is conceivable that LAL might be involved in RE metabolism, we examined whether mLAL exhibits RE hydrolase activity, the LAL specific inhibitor Lalistat 2 affects cellular RE levels, and LAL-deficient mice exhibit any changes in retinoid levels.

In this study, we show that LAL is the major acid RE hydrolase. This is evident from the complete inhibition of acid RE hydrolase activity in liver lysates upon addition of the LAL inhibitor Lalistat 2 (11), the largely reduced acid RE hydrolase activity in liver lysates of LAL-deficient mice, the accumulation of REs in endosomal/lysosomal-enriched fractions in the human hepatocyte-like HepG2 cells when incubated with Lalistat 2, and the increased RE accumulation in the duodenum and jejunum of LAL-deficient mice. Together, these findings indicate that LAL is the major acid RE hydrolase, required for efficient clearance of endocytosed REs and that, when absent (or inhibited), it cannot be compensated by other enzymes.

Studies in the 1980s and 1990s characterized the acid hydrolytic activity in the liver and found activities against chylomicron-contained REs, CEs, and TGs, whereas rates for TG hydrolysis were found to be highest (15). Furthermore, increasing concentrations of CEs were not found to compete for acid RE hydrolysis. Acid CE but not acid RE hydrolase activity of liver lysates was sensitive to micromolar concentrations of typical ionic halides (*e.g.* CaCl_2_) (15). These reports led to the conclusion that two distinct enzymes exist for the hydrolysis of CEs and REs in the liver. This view was further bolstered by studies of Blomhoff *et al.* (14) and Harrison *et al.* (13). The authors investigated the clearance of RE chylomicrons by injecting ^3^H-labeled RE containing chylomicrons into rats. They found that ^3^H radioactivity co-migrated time-dependently with the early endosomal fraction as well as the endoplasmic reticulum but to a much lesser extent or not at all with late endosomal/lysosomal fractions in hepatocytes (13, 14). The authors concluded that endocytosed REs are transferred from the endosomes directly to the endoplasmic reticulum, thereby bypassing lysosomes and thus not requiring acid RE hydrolase activity. Similarly, when murine J774 macrophages were incubated with ^3^H-labeled chylomicron REs, the radioactivity was found predominantly in early endosomes, to a much lesser extend in late endosomes/lysosomes, and was time-dependently converted to [^3^H]retinol. These findings indicated that endocytosed REs are already hydrolyzed in early endosomes (17) and are barely transferred to lysosomes, and thus do not require acid hydrolysis in lysosomes (18). However, and in clear contrast to previous reports, the results of this study show that LAL-deficient mice exhibit increased RE accumulation in the duodenum and jejunum, indicating that the clearance of REs also requires LAL activity. The observation that purified rabbit liver LAL hydrolyzes TGs and CEs at comparable rates (2) confirms our findings employing mLAL containing lysates. In addition, we show in *in vitro* activity assays that LAL also hydrolyzes RE at a rate similar to TGs and CEs, suggesting that LAL does not exhibit a strong preference for any of the neutral lipid ester substrates.

Our study confirms previous observations that LAL-deficient mice exhibit largely increased CE content in multiple tissues (*e.g.* 42.5-fold in livers of 8-months old LAL-deficient mice (4)). In comparison, we found that RE contents were significantly increased in the duodenum and jejunum but not in liver and lung, for example. In addition, the degree of RE accumulation was also much more moderate compared with that of CEs (*e.g.* 27-fold increased CE *versus* 2-fold increased RE content in duodenum of 6.5-month-old mice). This difference in the degree of CE *versus* RE accumulation in tissues of LAL-deficient mice is unlikely to reflect differences in the substrate preference of LAL because we found comparable rates in the hydrolysis of CE and RE. It is more feasible that REs are largely cleared in the circulation and early/late endosomes and that thus clearance of RE depends much less on the activity of LAL.

The observation that LAL-deficient mice exhibit increased RE levels in the duodenum (∼2-fold) and jejunum (∼7-fold) but not in the liver is, to a certain degree, unexpected. It is known that neutral lipid esters (including REs) are only taken up in the intestine after their complete hydrolysis involving transport proteins (19, 20). Thus, non-hydrolyzable retinyl ether is not taken up from the intestine (21), indicating that lipid esters are not endocytosed. Furthermore, Du *et al.* (4) reported that, in LAL-deficient mice, neutral lipids accumulate in proliferating macrophages in the lamina propria of intestinal villi. This observation suggests that increased RE content in the intestine of LAL-deficient mice might not derive from REs trapped in lysosomes of enterocytes but in macrophages. Consequently, these REs originate rather from the basolateral side of the intestine, the blood stream, or the lymph and not from the intestinal lumen. In addition, it has been reported that the genetic ablation of sterol *O-*acyltransferase 2 reduces CE accumulation in the intestine of LAL-deficient mice (∼50%) (22). This finding would be in line with the idea that neutral lipid esters, which accumulate in the intestine of LAL-deficient mice, derive from intracellular acyltransferase activity, are then cleared in lysosomes (*e.g.* hydrolysis of cytosolic lipid droplets via autophagy and requiring LAL (23)) and do not originate directly from endocytosed neutral lipids. Such an intracellular esterification process might be also valid for intestinal REs and may involve LRAT/acyl-CoA:retinol acyltransferase activity. The observation, however, that LAL-deficient mice exhibited reduced post-prandial circulating RE levels upon RE gavage suggests that LAL may also be required for the availability of nutritional neutral lipid esters absorbed from the luminal side of the intestine.

This finding that LAL-deficient mice exhibit reduced post-prandial circulating RE levels (upon RE bolus) might be rather interpreted as impaired fat absorption. In line, LAL-deficient mice have been reported to exert ∼1.5-fold increased fecal neutral sterol excretion (12). In our study, we have attempted to measure retinoid excretion but were not able to detect any retinoids in the feces of either WT or LAL-deficient mice. To circumvent issues such as detection limit or instability of retinoids during gut transit, a more sophisticated technique such as the use of radiolabeled retinoids would be required. However, the fact that, in a recent study, 13% of human CE storage disease patients suffer from chronic diarrhea (24) could be also indicative of intestinal malabsorption.

An apparent difference in disturbed CE *versus* RE homeostasis of LAL-deficient mice is evident from the observation that CE accumulates in all tissues investigated, whereas RE levels are decreased by ∼30% in the lung and liver (note that the total RE content of livers of LAL-deficient mice was increased compared to that of WT mice because the livers of LAL-deficient mice were 3.6-fold enlarged). Decreased RE content in the lung and liver might be explained by decreased nutritional availability of vitamin A, as evident from decreased post-prandial circulating REs. The observation, however, that CE accumulate manyfold in the liver and lung of LAL-deficient mice seemingly disproves this concept. In any case, it is apparent from the lipid phenotype of the LAL-deficient mouse that LAL is essential for CE but not for RE homeostasis. The reason for this discrepancy is unexplained.

In summary, we conclude that LAL is the major acid RE hydrolase. Deficiency of LAL results in RE accumulation in the intestine (duodenum and jejunum) and a reduced post-prandial circulating RE level, indicative of impaired nutritional availability of REs. Thus, LAL is required for functional vitamin A homeostasis but, when absent, does not provoke vitamin A deficiency.

## Author Contributions

L. G. conducted most of the experiments. T. O. E. analyzed the lipid species by MS. U. T., K. A. Z., and C. L. contributed to the animal experiments. M. R. cloned the LAL construct. C. Y., H. D., and D. K. provided the LAL-deficient mice. L. G., U. T., and A. L. wrote the manuscript. T. M., B. R., G. H., R. Zechner, P. F., D. K., and R. Zimmermann discussed the manuscript. All authors contributed intellectually to the manuscript.

## Footnotes

2 The abbreviations used are:

LAL: lysosomal acid lipase
CE: cholesteryl ester
RE: retinyl ester
TG: triglyceride
ROH: retinol
mLAL: murine lysosomal acid lipase
RP: retinyl palmitate
FD: fluorescence detection
PC: phosphatidylcholine
PI: phosphatidylinositol
BMP: *sn*-1,*sn*-1′ bis(monoacylglycero)phosphate
mHSL: murine hormone-sensitive lipase
LRAT: lecithin:retinol acyltransferase
RS: retinyl stearate
M/M: moles per moles.

## References

[1] Sheriff S., Du H., Grabowski G. A. (1995) Characterization of lysosomal acid lipase by site-directed mutagenesis and heterologous expression. J. Biol. Chem. 270, 27766–27772 7499245 10.1074/jbc.270.46.27766

[2] Imanaka T., Amanuma-Muto K., Ohkuma S., and Takano T. (1984) Characterization of lysosomal acid lipase purified from rabbit liver. J. Biochem. 96, 1089–1101 6520114 10.1093/oxfordjournals.jbchem.a134926

[3] Porto A. F. (2014) Lysosomal acid lipase deficiency: diagnosis and treatment of Wolman and cholesteryl ester storage diseases. Pediatr. Endocrinol. Rev. 12, 125–132 25345094

[4] Du H., Heur M., Duanmu M., Grabowski G. A., Hui D. Y., Witte D. P., and Mishra J. (2001) Lysosomal acid lipase-deficient mice: depletion of white and brown fat, severe hepatosplenomegaly, and shortened life span. J. Lipid Res. 42, 489–500 11290820

[5] Du H., Duanmu M., Witte D., and Grabowski G. A. (1998) Targeted disruption of the mouse lysosomal acid lipase gene: long-term survival with massive cholesteryl ester and triglyceride storage. Hum. Mol. Genet. 7, 1347–1354 9700186 10.1093/hmg/7.9.1347

[6] Fouchier S. W., and Defesche J. C. (2013) Lysosomal acid lipase A and the hypercholesterolaemic phenotype. Curr. Opin. Lipidol. 24, 332–338 23652569 10.1097/MOL.0b013e328361f6c6

[7] Hoeg J. M., Demosky S. J. Jr., Pescovitz O. H., and Brewer H. B. (1984) Cholesteryl ester storage disease and Wolman disease: phenotypic variants of lysosomal acid cholesteryl ester hydrolase deficiency. Am. J. Hum. Genet. 36, 1190–1203 6097111 PMC1684644

[8] Pollak N. M., Schweiger M., Jaeger D., Kolb D., Kumari M., Schreiber R., Kolleritsch S., Markolin P., Grabner G. F., Heier C., Zierler K. A., Rülicke T., Zimmermann R., Lass A., Zechner R., and Haemmerle G. (2013) Cardiac-specific overexpression of perilipin 5 provokes severe cardiac steatosis via the formation of a lipolytic barrier. J. Lipid Res. 54, 1092–1102 23345410 10.1194/jlr.M034710 PMC3605985

[9] Joutti A., Vainio P., Brotherus J. R., Paltauf F., and Kinnunen P. K. (1981) The active site and the phospholipid activation of rat liver lysosomal lipase are not stereospecific. Chem. Phys. Lipids. 29, 235–239 7296725 10.1016/0009-3084(81)90054-2

[10] Ström K., Gundersen T. E., Hansson O., Lucas S., Fernandez C., Blomhoff R., and Holm C. (2009) Hormone-sensitive lipase (HSL) is also a retinyl ester hydrolase: evidence from mice lacking HSL. FASEB J. 23, 2307–2316 19246492 10.1096/fj.08-120923

[11] Hamilton J., Jones I., Srivastava R., and Galloway P. (2012) A new method for the measurement of lysosomal acid lipase in dried blood spots using the inhibitor Lalistat 2. Clin. Chim. Acta 413, 1207–1210 22483793 10.1016/j.cca.2012.03.019

[12] Aqul A., Lopez A. M., Posey K. S., Taylor A. M., Repa J. J., Burns D. K., and Turley S. D. (2014) Hepatic entrapment of esterified cholesterol drives continual expansion of whole body sterol pool in lysosomal acid lipase-deficient mice. Am. J. Physiol. Gastrointest. Liver Physiol. 307, G836–G847 25147230 10.1152/ajpgi.00243.2014 PMC4200320

[13] Harrison E. H., Gad M. Z., and Ross A. C. (1995) Hepatic uptake and metabolism of chylomicron retinyl esters: probable role of plasma membrane/endosomal retinyl ester hydrolases. J. Lipid Res. 36, 1498–1506 7595074

[14] Blomhoff R., Eskild W., Kindberg G. M., Prydz K., and Berg T. (1985) Intracellular transport of endocytosed chylomicron [^3^H]retinyl ester in rat liver parenchymal cells. Evidence for translocation of a [^3^H]retinoid from endosomes to endoplasmic reticulum. J. Biol. Chem. 260, 13566–13570 2414283

[15] Mercier M., Forget A., Grolier P., and Azais-Braesco V. (1994) Hydrolysis of retinyl esters in rat liver: description of a lysosomal activity. Biochim. Biophys. Acta 1212, 176–182 8180243 10.1016/0005-2760(94)90251-8

[16] Eichmann T. O., Grumet L., Taschler U., Hartler J., Heier C., Woblistin A., Pajed L., Kollroser M., Rechberger G., Thallinger G. G., Zechner R., Haemmerle G., Zimmermann R., and Lass A. (2015) ATGL and CGI-58 are lipid droplet proteins of the hepatic stellate cell line HSC-T6. J. Lipid Res. 56, 1972–1984 26330055 10.1194/jlr.M062372 PMC4583087

[17] Hagen E., Myhre A. M., Tjelle T. E., Berg T., and Norum K. R. (1999) Retinyl esters are hydrolyzed in early endosomes of J774 macrophages. J. Lipid Res. 40, 309–317 9925661

[18] Harrison E. H. (1993) Enzymes catalyzing the hydrolysis of retinyl esters. Biochim. Biophys. Acta 1170, 99–108 8399348 10.1016/0005-2760(93)90058-h

[19] Reboul E. (2013) Absorption of vitamin A and carotenoids by the enterocyte: focus on transport proteins. Nutrients 5, 3563–3581 24036530 10.3390/nu5093563 PMC3798921

[20] Iqbal J., and Hussain M. M. (2009) Intestinal lipid absorption. Am. J. Physiol. Endocrinol. Metab. 296, E1183–E1194 19158321 10.1152/ajpendo.90899.2008 PMC2692399

[21] Weng W., Li L., van Bennekum A. M., Potter S. H., Harrison E. H., Blaner W. S., Breslow J. L., and Fisher E. A. (1999) Intestinal absorption of dietary cholesteryl ester is decreased but retinyl ester absorption is normal in carboxyl ester lipase knockout mice. Biochemistry 38, 4143–4149 10194330 10.1021/bi981679a

[22] Lopez A. M., Posey K. S., and Turley S. D. (2014) Deletion of sterol *O-*acyltransferase 2 (SOAT2) function in mice deficient in lysosomal acid lipase (LAL) dramatically reduces esterified cholesterol sequestration in the small intestine and liver. Biochem. Biophys. Res. Commun. 454, 162–166 25450374 10.1016/j.bbrc.2014.10.063 PMC4312202

[23] Ouimet M., Franklin V., Mak E., Liao X., Tabas I., and Marcel Y. L. (2011) Autophagy regulates cholesterol efflux from macrophage foam cells via lysosomal acid lipase. Cell Metab. 13, 655–667 21641547 10.1016/j.cmet.2011.03.023 PMC3257518

[24] Burton B. K., Deegan P. B., Enns G. M., Guardamagna O., Horslen S., Hovingh G. K., Lobritto S. J., Malinova V., McLin V. A., Raiman J., Di Rocco M., Santra S., Sharma R., Sykut-Cegielska J., Whitley C. B., et al (2015) Clinical features of lysosomal acid lipase deficiency. J. Pediatr. Gastroenterol. Nutr. 61, 619–625 26252914 10.1097/MPG.0000000000000935 PMC4645959

